# Physicochemical properties and bioturbation analysis of high-temperature Daqu by functional microflora

**DOI:** 10.3389/fmicb.2025.1601675

**Published:** 2025-06-26

**Authors:** Tingyan Xu, Anqin Zhu, Shuting Zhong, Yunsheng Wang, Chao Wang, Jin Zhang, Yincui Chen, Rui Wang, Chuanbo Zhang

**Affiliations:** ^1^Laboratory of Microbial Resources and Industrial Application, College of Life Sciences, Guizhou Normal University, Guiyang, China; ^2^College of Resources and Environmental Engineering, Guizhou University, Guiyang, China; ^3^Department of Brewing, Guizhou Guijiu Group Limited Liability Company, Xiuwen, China; ^4^Guizhou Vocational College of Agriculture, Qingzhen, China

**Keywords:** synthetic functional microflora, fortified Daqu, fermentation, microbial diversity, assembly process

## Abstract

High-temperature Daqu (HTD) initiates the solid-state fermentation of sauce-aroma Baijiu by introducing essential microorganisms and enzymes, determining its unique flavor and quality. While biofortification with functional strains enhances HTD quality, the ecological dynamics and mechanisms of HTD fermentation influenced by synthetic functional microflora (SFM) remain unclear. Here, we prepared three different types of SFM inoculated into HTD, and studied their effects during spontaneous HTD fermentation. The results showed that all three SFM significantly increased the liquefaction power of the HTD, which the SG Daqu with the addition of yeast and *Eurotium amstelodami* synthetic flora were the most effective, and also had a significant increase in fermentation and esterification power. All three SFM increased the average relative abundance of *Bacillus* and decreased the relative abundance of *Lactobacillus* and *Weissella*, and enhanced the stability of the fungal community. The significant bioturbation effect of SFM on the bacterial community of HTD was predominantly observed during the 3-day fermentation period, while its substantial impact on the fungal community manifested during the 9-day fermentation. Meanwhile, the SFM influenced the community assembly pattern of HTD and stability of the network. Notably, PICRUSt2 revealed that the addition of SFM increased the potential ability of HTD to utilize energetic substances such as starch for metabolism and energy conversion, to generate ethanol and esters, and to facilitate ethanol metabolism. Overall, our work elucidated the regulatory mechanism of SFM on the longitudinal characteristics of microbial communities in the HTD fermentation stage, and provided a theoretical basis for further research on SFM to enhance the quality of HTD.

## 1 Introduction

Sauce-aroma Baijiu, one of the most popular liquors in China with a history spanning thousands of years, contains a wide range of flavor compounds that are beneficial to the human body (Liu and Sun, 2018; Wang et al., 2018). As a unique saccharification starter necessary for the production of sauce-aroma Baijiu, high-temperature Daqu (HTD) enriches a variety of microorganisms from an open environment. It provides functional microorganisms and functional enzymes as well as unique volatile components and precursors which are of great importance in the production process of sauce-aroma Baijiu (Huang et al., 2017b; Jin et al., 2017; Jin et al., 2019; Li H. et al., 2023; Zhang et al., 2021). Since the fermentation process of HTD occurs in a naturally open environment, making the formation and stability of its micro-ecological system highly dependent on the Muqu and environmental microorganisms (Huang et al., 2017a; Zhou et al., 2024). Microbial interactions are strongly influenced by endogenous bioheat from microbial metabolism and abiotic factors such as moisture, acidity, and starch (Fu G. et al., 2021; Ma et al., 2022; Xiao et al., 2017). The microbial succession during the fermentation stage occurs spontaneously and develops independently of human intervention, which limits the occurrence and effectiveness of synergistic effects among microbial communities. As a result, ensuring the consistency of quality and functional expression in HTD becomes challenging (Li et al., 2020). On the other hand, the microecosystem of the brewing environment is influenced by various production conditions, resulting in the accumulation of both beneficial and harmful microorganisms within the naturally occurring microbial community. This dynamic contributes to the inconsistent quality of HTD and elevates production costs. Hence, optimizing the microbial community structure and enhancing the quality of HTD is of paramount importance.

With the increasing consumer demand for safe, healthy and high-quality functional foods, optimizing fermentation processes to improve the taste and quality of fermented foods has become a bottleneck (Jin et al., 2024; Shiferaw Terefe and Augustin, 2019). Functional microorganisms play an important role in improving the safety and quality of fermented foods by positively affecting the microbial community and material metabolism during the fermentation process. Notably, co-fermentation with multiple microorganisms has emerged as a practical strategy for utilizing synthetic functional microorganisms (SFM) in food fermentation processes (Cui et al., 2018; Gao et al., 2022; Yin et al., 2024), and has been involved in the production of fermented foods such as vinegar, kimchi, soy sauce, and Baijiu (Fu et al., 2022; Lee et al., 2011; Liang et al., 2019; Tang Q. et al., 2024). In recent years, the feasibility of improving the quality of Daqu through bio-fortification with inoculated functional microorganisms has been demonstrated. This approach also shows potential for reducing safety risks and enhancing the flavor and quality of fermented foods (Tian et al., 2022; Zhang et al., 2024). Previous studies have shown that fortification of Daqu fermentation with *Bacillus velezensis* and *Bacillus subtilis* can improve the liquefaction, saccharification, and esterification power of Daqu fermentation (He et al., 2019b). The application of biofortified Daqu in Baijiu brewing increased the content and diversity of esters and aromatic compounds in Baijiu samples (He et al., 2020). Mu et al. (2023c) found that biofortification of Daqu increased the content of aroma substances such as ethyl hexanoate, 4-methylphenol, dimethyl trisulfide, and ethyl butanoate. Inoculation of Various Yeasts into the Daqu decreased ethanol and aldehyde content, while increasing ester content, altering microbial community structure, and metabolic activity (Li et al., 2020; Pang et al., 2023). This evidence suggested that biofortification strategies have great potential for improving the quality of Daqu and the flavor of Baijiu.

Next,-generation sequencing, molecular biology, and culture-dependent technologies have been widely used to investigate the structural composition of the microbial community, process parameters, microbial succession, and physicochemical characteristics of HTD (Wen et al., 2023; Xia et al., 2022a; Yi et al., 2019), and laying a theoretical foundation for the study of functional strains or flora biofortification of Daqu. However, research is still lacking on how the integration of SFM affects the longitudinal dynamics and ecological characteristics of microbial communities during the spontaneous fermentation phase of HTD. Moreover, the mechanisms by which SFM enhances HTD functionality remain unclear. Our previously reported work has illustrated that *Eurotium amstelodami*, yeast and *Bacillus* isolated from high-temperature Daqu have excellent fermentation potential for improving the quality and yield of fermentation products (Chen et al., 2022). These strains are capable of forming symbiotic relationships and producing secondary metabolites (Wang et al., 2023a). This study innovatively used *E. amstelodami* with excellent fermentation potential as the core strain for making fortified Daqu, which has never been reported in previous studies. In this work, we developed three SFMin an open environment (EG: inoculation with synthetic flora of *E. amstelodami*; SG: inoculation with synthetic flora of *E. amstelodami* with yeasts; MG inoculation with synthetic flora of *E. amstelodami*, yeasts, and *Bacillus*). These strains were isolated from high quality sauce-flavor Baijiu HTD and Jiupei produced in Renhuai city, Guizhou Province. We systematically investigated their impact on the quality of HTD. Furthermore, we employed high-throughput sequencing technology to analyze the community structure and dynamic changes of microorganisms throughout the fermentation process.

## 2 Materials and methods

### 2.1 Inoculation experiments

The test strains *Eurotium amstelodami* DW-7, DP-25, DP-35, *Wickerhamomyces anomalus* DY-Z1, *Bacillus velezensis* DL-7, *Bacillus amyloliquefaciens* DP-2, *Heyndrickxia coagulans* DA-6, and *Bacillus licheniformis* DC-3 were isolated from high-quality sauce-flavor Baijiu HTD produced in Renhuai city, Guizhou Province. *Saccharomyces cerevisiae* JW-1, *Pichia pastoris* JW-26 and *Hansenula hansenii* JY-2 were isolated from sauce-aroma Baijiu Jiupei. These isolates were identified by molecular biology and preserved in the Research Laboratory of Microbial Resources and Industrial Application of Guizhou Normal University, Guizhou Province, China. The production process and conditions of biofortified Daqu are identical to those of traditional Daqu, and both were manufactured in the Daqu solid-state fermentation chamber of Guizhou Guijiu Group Co., Ltd. (Xiuwen County, Guizhou Province, China). Spore suspensions and bacterial suspensions were prepared by the method of Chen et al. (2020). Briefly, *E. amstelodam*i, yeast, and *Bacillus* strains were inoculated into PDA medium, respectively. *Bacillus* was incubated at 37°C in an incubator for 24 h, while *E. amstelodami* and yeast were incubated at 30°C in an incubator at 30°C for 48 h and 5 d, respectively. *Bacillus* and yeasts were eluted with sterile water to prepare bacterial suspensions, and bacterial concentrations were adjusted to approximately 2 × 10^6^ CFU/mL. *E. amstelodami* was scraped and placed in a triangular vial containing sterile water and glass beads, shaken at 180 rpm/min for 30 min until the spores were dispersed homogeneously, and the concentration was adjusted to 2 × 10^6^ CFU/mL to make a spore suspension.

**Figure 1:**
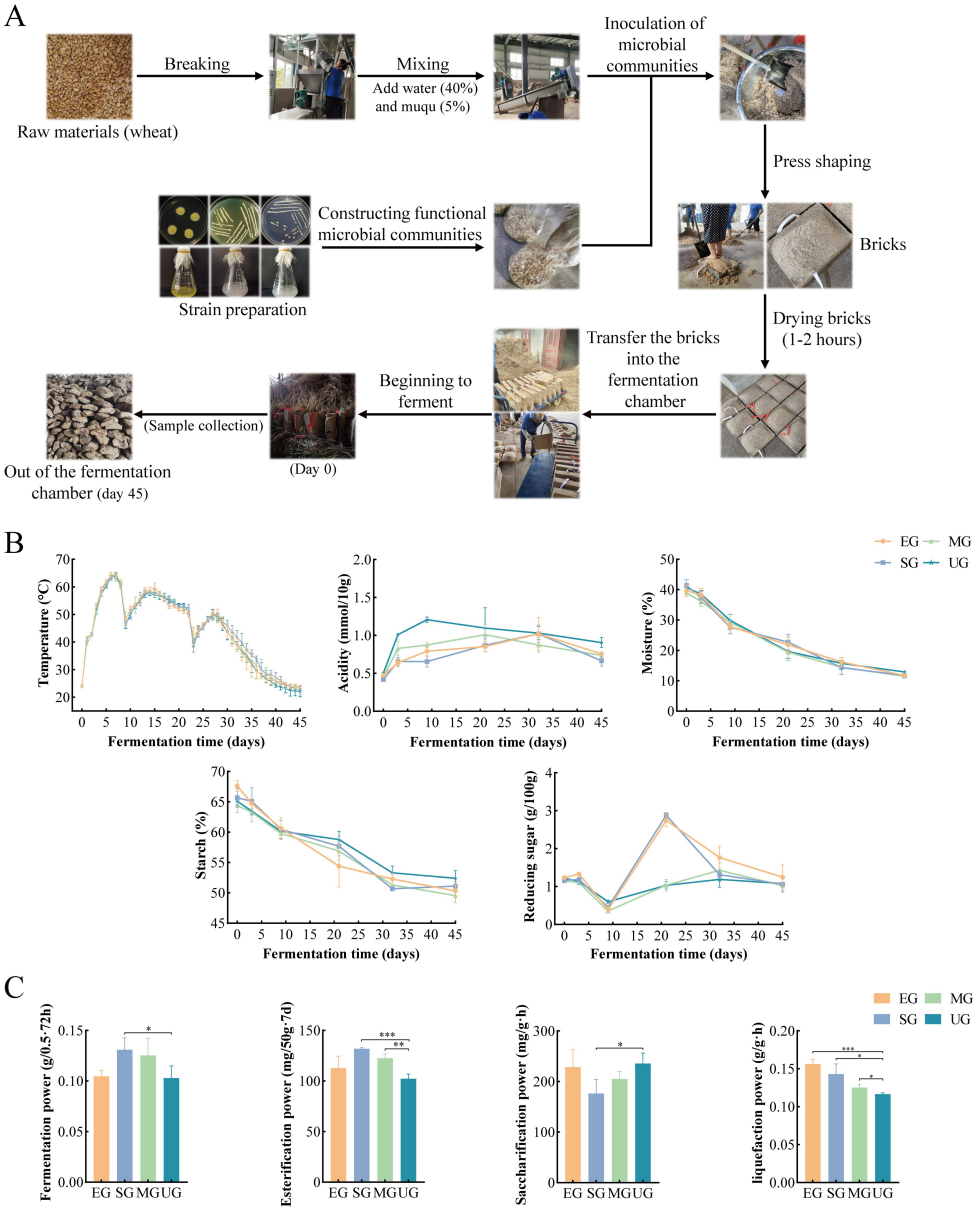
Sketch of the fortified HTD production process **(A)**. Changes in physicochemical parameters during the fermentation stage of HTD **(B)**. Changes in enzyme activity at the end of HTD fermentation **(C)**. Significant differences are indicated as follows: *p < 0.05, **p < 0.01, and ***p < 0.001. EG, SG, and MG represent fortified HTD, respectively, UG represent traditional HTD.

For the preparation of biofortified HTD, all four groups of HTD were inoculated with Muqu, where UG served as a control group. However, EG group was obtained by inoculation with synthetic flora of *E. amstelodami*. SG group was obtained by inoculation with synthetic flora of *E. amstelodami* with yeasts (with a seeding culture ratio of 1:1, v/v). MG group was obtained by inoculation with synthetic flora of *E. amstelodami*, yeasts, and *Bacillus* (with a seeding culture ratio of 1:1:1, v/v). The strain was inoculated at 3% (v/v) and all four groups of Daqu were inoculated with Muqu. The production process of biofortified HTD is shown in Figure 1A. The wheat was crushed and combined with 40% (w/w) water and 5% (w/w) Muqu. After the strain was added and thoroughly mixed, the mixture was loaded into a mold measuring 40 × 25 × 11 cm and manually pressed into a rectangular brick. After drying the bricks for 1–2 h, they were transferred to the fermentation chamber, stacked in layers, with each brick separated by straw, and incubated for about 35–45 days (the incubation time was adjusted by an experienced brewer, and was slightly longer in autumn and winter than in spring and summer).

### 2.2 Sampling

To investigate the effects of different SFM in an open fermentation environment on various physicochemical indexes and microbial community dynamics during the fermentation stage of HTD. Samples were collected at different periods during the fermentation stage of the fermentation chamber at 0 d (period A), 3 d (period B), 9 d (period C, primary turnover of the Daqu), 23 d (period D, secondary turnover of the Daqu), 35 d (period E), and 45 d (period F). Sampling was carried out and a total of 72 samples (three replicates for each sample) were obtained for subsequent analyses.

### 2.3 Environmental and physicochemical property analysis

A precision digital thermometer was placed in the center of the quartz embryo to monitor the temperature in real time during the making of Daqu and recorded daily. Moisture was determined by the constant weight method at 105°C (He et al., 2019a). Determination of total acidity of Daqu concerning the method of Dong et al. (2022). The rest of the physicochemical indexes were determined according to the industry standard QB/T4257-2011 (General Methods of Analysis for Daqu of China), and all the samples were repeated 3 times.

### 2.4 Genomic DNA extraction, amplification and sequencing

Microbial community genomic DNA was extracted from 72 Daqu samples using the E.Z.N.A.^®^ soil DNA Kit (Omega Bio-tek, Norcross, GA, United States) according to the manufacturer’s instructions. The concentration and purity of DNA were determined by a NanoDrop 2000 UV–vis spectrophotometer, 1% Agarose Gel Electrophoresis for DNA Quality Detection. Bacterial 16S rRNA gene V3–V4 hypervariable regions were amplified using universal primers 338F and 806R, while fungal ITS1-ITS2 regions of the ITS rRNA gene were targeted with primers ITS1F and ITS2R, respectively, using an ABI GeneAmp^®^ 9,700 PCR thermocycler (Applied Biosystems, San Francisco, CA, United States). Please consult supporting information for the detailed methodology. Purified amplicons were pooled in equimolar and paired-end sequenced on an Illumina MiSeq PE300 platform/NovaSeq PE250 platform (Illumina, San Diego, CA, United States) according to the standard protocols by Majorbio Bio-Pharm Technology Co., Ltd. (Shanghai, China). The raw sequencing data have been deposited in the NCBI Sequence Read Archive (SRA) under BioProject accession numbers PRJNA1232965 (bacterial) and PRJNA1233011 (fungal).

### 2.5 Processing of sequencing data

The data of each sample were identified according to the index sequence and saved in FASTQ format, while the paired reads were spliced into one sequence based on the overlap between PE reads, and quality control was performed on the read quality and merging effect. Operational taxonomic units (OTUs) were clustered with a 97% similarity cutoff using UPARSE (version 11), and chimeric sequences were identified and removed using UCHIME (Edgar, 2013). The taxonomy of each OTU representative sequence was analyzed by RDP Classifier (version 11.5) against the bacterial 16S rRNA database (SILVA 138.2) and fungal ITS database (UNITE 10.0) under the confidence threshold of 0.7.

### 2.6 Statistical analysis

Statistical analysis of this study was carried out using Excel (version 2019) for data processing. GraphPad Prism (version 8.0.2) was used for graphing the physicochemical properties. Unless stated otherwise, all analyses presented in the text were performed utilizing the R environment (version 3.3.1). Linear discriminant analysis (LDA) effect size (LEfSe) analysis was used to identify the significantly abundant bacterial and fungal taxa (from phylum to genus level) among the different groups. Co-occurrence network analysis was conducted using R software (version 4.3.1), and the interaction between network nodes was visualized using Gephi software (version 0.10.1). Based on the icamp package (version 1.5.12) of the R environment (version3.3.1), the beta nearest-taxon index (βNTI) is calculated using the null model (*n* = 999). βNTI was used to quantify the phylogenetic turnover between the four groups of HTD microbial communities as it was able to compare the mean nearest taxonomic unit distances (βMNTD) between the actual observed communities with the expected values from the null model simulations. RC_bray_ (Raup-Crick index combined with Bray-Curtis distance) generates the desired Bray–Curtis dissimilarity distribution by modelling the randomness of community species distributions to construct a null model and comparing it with actual observations. We performed several calculations using the βNTI and the Raup-Crick metric based on Bray–Curtis dissimilarity (RC_bray_) to distinguish the relative roles of deterministic and stochastic processes. Subsequently, the RC_bray_ was utilized to assess the relative contributions of five ecological processes, namely Homogeneous selection (HoS), Heterogeneous selection (HeS), Dispersal limitation (DL), Homogeneous dispersal (HD), and Drift (DR), to the assembly of the HTD microbial community. Canoco 5.0 software was used to complete the redundancy analysis (RDA). PICRUSt2 predicts the functional gene profiles of microorganisms by constructing phylogenetic trees of sample microbial communities and combining them with functional annotations from reference genome databases. The prediction of microbial functions was conducted using PICRUSt2, which compares microbial functions to the KEGG pathway database.

## 3 Results

### 3.1 The production process of fortified HTD and the changes in its physicochemical properties

A sketch of the SFM biofortified HTD production process is shown in the Figure 1A. The trends of temperature, moisture, and starch content of biofortified EG, SG, and MG HTD were consistent with those of control UG (Figure 1B). However, the acidity and starch content of EG, SG, and MG was lower than that of UG at 9–45 days. The reducing sugar content of EG and SG was higher than that of UG and MG at 9–35 days. The acidity of EG, SG, and MG was higher than that of UG. In addition, the esterification power, saccharification power, liquefaction power, and fermentation power of HTD at the end of fermentation were measured and used to evaluate the enzymatic function of HTD after the addition of SFM (Figure 1C). The fermentation power of UG was 0.103 (g/0.5 g·72 h), SG was 0.131 (g/0.5 g·72 h) significantly higher than that of UG (*p* < 0.05), and MG was 0.125 (g/0.5 g·72 h), which was also higher than that of UG. The saccharification power of SG was 176.49 (mg/g·h) significantly lower than that of UG (*p* < 0.05). The esterification power was 102.26 (mg/50 g·7d) for UG, while it was significantly higher for SG (131.91 mg/50 g·7d) and MG (122.61 mg/50 g·7d) compared to UG (*p* < 0.01). The liquefaction power of UG was significantly lower than that of the other three groups, with EG having the highest liquefaction power of 0.157 (g/g·h), followed by SG and MG.

### 3.2 Effects of different SFM on microbial alpha diversity, community structure composition, and dynamic successional changes during the fermentation stage of HTD

We finally obtained 3,860,401 high-quality 16S rRNA and 5,059,878 ITS gene amplicon sequences through Illumina MiSeq sequencing. The total base counts of these sequences were 1,177,310,333 bp and 1,643,069,256 bp, with average lengths of 425 bp and 232 bp, respectively. They were subsequently clustered into 717 and 361 OTUs (Supplementary Tables S1, S2). The bacterial and fungal coverage index ranged from 99.89 to 99.97%, indicating that the sequencing results can comprehensively and accurately reflect the microbial diversity information in the HTD samples (Supplementary Table S3). Meanwhile, the dilution curves indicate that the amount of sequencing data in this study is reasonable and the depth of sequencing is suitable to represent the diversity of microorganisms in the samples (Supplementary Figures S1A,B).

**Figure 2:**
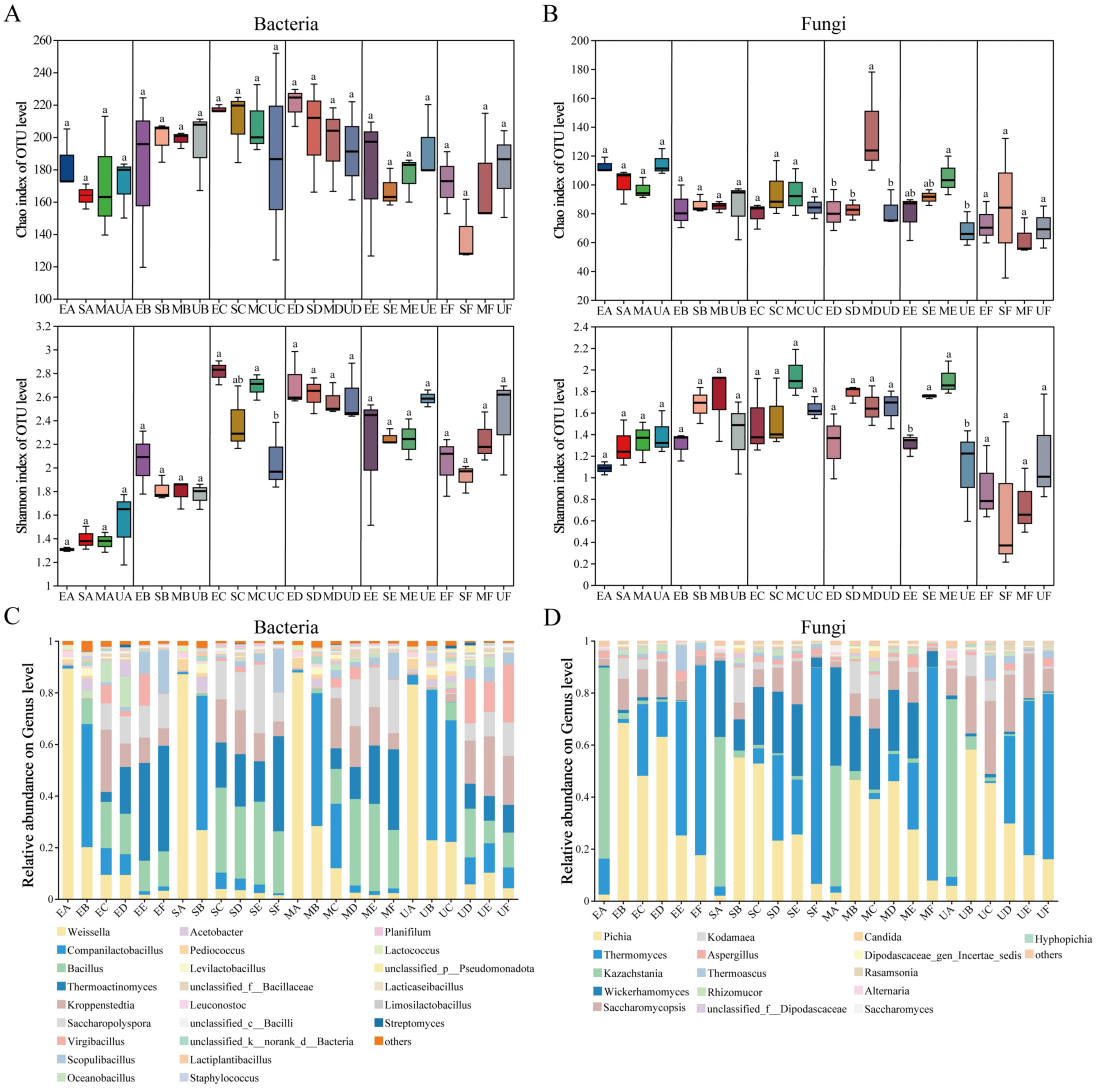
Changes in microbial community *α*-diversity index during various fermentation periods of four groups of HTD samples, **(A)** Bacterial Chao and Shannon indexes, **(B)** Fungal Chao and Shannon indexes. The relative abundance trend of the microbial community at the genus level during the fermentation process of each group of HTD samples was demonstrated, showing only the genera with an average relative abundance >0.1%, **(C)** bacteria and **(D)** fungi.‌‌ Significant differences were observed between sample groups denoted by different letters (*p* < 0.05).

Throughout the entire fermentation process of Daqu, we observed consistent trends in the Chao index and Shannon index for both bacteria and fungi across different treatment groups (Figures 2A,B). Interestingly, the bacterial Chao index of SG and MG reached its maximum in C period, while EG and UG reached its maximum only in D period. The bacterial Shannon index of EG and MG groups were significantly higher than that of UG group in C period. However, no significant differences were observed among the different treatments in E-F periods. Fungal Chao index of all four groups of Daqu underwent a decrease in the A-B periods. Interestingly, the Chao index of MG was significantly increased (*p* < 0.05) in the D-E periods. Meanwhile, the Shannon index of MG and SG also increased in the B-E periods.

To further investigate the effects of bioturbation of SFM on the fermentation stage of HTD, we evaluated the heterogeneity of microbial communities in four groups of Daqu as well as the dynamic successional changes at the phylum and genus levels. Supplementary Figures S1C,D showed that different SFM at the phylum level exerted different impacts on the bacterial and fungal communities of HTD. In genus level, a total of 240 bacterial and 156 fungal were annotated, respectively. Our results indicated that the bacterial communities of the four groups of HTD were similar in period A (Figure 2C), but the average relative abundance (RA) of the bacterial communities of EG, SG, and MG differed significantly from that of UG as fermentation progressed. *Weissella* as the dominant bacterial genus showed a gradual decrease with fermentation in all four groups of HTD, with small differences in the RA of UG (22.81%), EG (20.11%), SG (26.75%), and MG (28.29%) during period B. However, in period C, the RA of *Weissella* was significantly reduced in the EG (9.40%), SG (3.90%), and MG (11. 95%) groups compared to the UG group (22.15%). This finding suggests that the addition of SFM decreased the RA of *Weissella* in Daqu to varying degrees, with the most pronounced effect occurring during period C. Similarly, *Companilactobacillus*, as the dominant bacterial genus in the four groups of Daqu during the fermentation stage, increased rapidly in the B period and decreased significantly in periods D-F. Notably, the RA of *Companilactobacillus* reached 58.26% in UG during the B period, which was higher than that of EG (47.70%), SG (52.05%), and MG (51.51%), and similarly slightly higher than that of EG, SG, and MG during C-F periods. Meanwhile, *Bacillus* began to proliferate during period C, with the RA in the EG (17.91%), SG (32.94%), and MG (13.50%) groups were higher than that of UG group (7.00%). Notably, in the MG group, the RA of *Bacillus* increased to 33.52% in D period, which was higher than that of UG (18.50%). The fungal communities of HTD varied at the genus level (Figure 2D). As the dominant fungal genus, the RA of *Pichia* was the highest in all four groups of HTD, showing an increasing and then decreasing trend throughout the fermentation stage. In period A, the RA of *Wickerhamomyces* showed significant differences among the four groups, with UG at 1.43%, and EG, SG, and MG at 0.73, 29.26, and 37.80%, respectively. During periods B-E, the RA in the UG and EG groups remained relatively stable compared to period A, while the RA in the SG and MG groups declined slightly but remained at higher levels. However, a sharp decline in the RA of SG and MG was observed in period F. As the dominant fungal genus, the RA of *Thermomyces* in the four groups of HTD decreased sharply and was less than 1% in period B. Notably, the RA of *Thermomyces* in EG, SG, and MG increased rapidly in period C, and the highest increase was observed in EG (27.54%). In contrast, the RA of *Thermomyces* in the UG group remained below 1%. In the F period, *Thermomyces* became the absolute dominant fungal genus in all four groups of HTD (63.52, 72.87, 83.16 and 81.96% in UG, EG, SG, and MG, respectively). Interestingly, we observed that *Thermoascus*, *Aspergillus*, *Kodamaea*, and *Rhizomucor* were reduced to 1% in the UG group during period E, while these fungal in the EG, SG, and MG were not changed as drastic as in UG. These results indicated that different SFM exerted significant bioturbation effects on the microbial community of HTD during the fermentation stage.

### 3.3 Characterization of the phases of bioturbation of SFM during the fermentation stage of HTD and the periods when functional microorganisms play an important role

**Figure 3:**
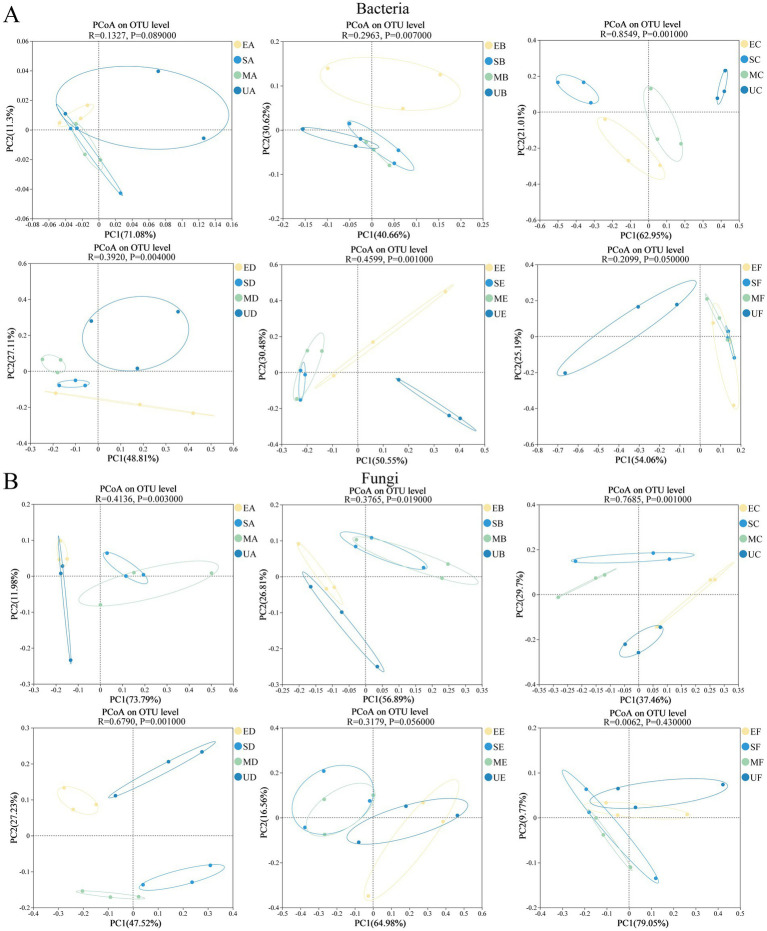
Principal co-ordinates analysis of microbial community during the fermentation process of four groups of HTD, **(A)** bacteria and **(B)** fungi.

PCoA analysis and ANOSIM similarity analysis were utilized to assess and examine the stage characteristics of differences in microbial structure of HTD caused by different SFM. There were no significant differences in the bacterial communities among the four HTD groups during period A (ANOSIM: R = 0.1327, *p* = 0.0890) (Figure 3A). However, significant differences emerged between the HTD bacterial communities in the groups during periods B–E. We observed that the direction of bacterial succession in EG, SG, and MG was similar, while it was significantly different from the bacterial community in UG in the period F. In contrast, significant differences were observed between the groups of fungal communities during periods A–D. However, the structure of the HTD fungal communities in the four groups became similar during periods E–F (Figure 3B).

**Figure 4:**
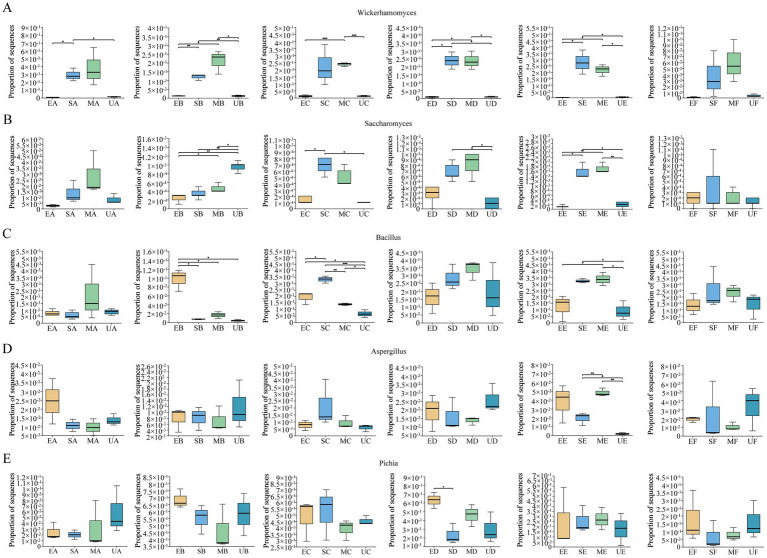
Periods when functional microorganisms in SFM exert important bioturbation effects on the microbial community of HTD, **(A)** *Wickerhamomyces*, **(B)** *Saccharomyces*, **(C)** *Bacillus*, **(D)** *Aspergillus* and **(E)** *Pichia*. Kruskal-Wallis H test was used to compare the difference of abundance between four groups. Significant differences are indicated as follows: *p < 0.05, **p < 0.01, and ***p < 0.001.

Functional microorganisms inoculated into four groups of HTD were analyzed for significant differences at the genus level using the Kruskal-Wallis H test and revealed the period during which they acted on HTD. The proportion of sequences (PS) of *Wickerhamomyces* was significantly higher in the SG group compared to the UG and EG groups (*p* < 0.05), while other functional microorganisms did not show significant differences among the four groups (Figure 4). In period B, the PS of *Wickerhamomyces* increased significantly in both SG and MG groups and remained higher compared to UG and EG (*p* < 0.05). In period C, the PS in the MG group was higher than in both EG and UG groups (*p* < 0.001). During periods D-E, the PS of *Wickerhamomyces* in SG and MG continued to be significantly higher than in UG and EG (*p* < 0.05). These results suggest that *Wickerhamomyces* had a pronounced effect on the microbial environment of the MG and SG groups starting in period B, which persisted through period E (Figure 4A). The PS of *Saccharomyces* were higher in UG than EG, SG, and MG (*p* < 0.05) at period B. Notably, The PS of *Saccharomyces* in SG was higher than UG and EG (*p* < 0.05) during periods C–E. This result suggests that the addition of SFM with *Saccharomyces* may have an important bioturbation effect on HTD microbial community in C–E periods (Figure 4B). The PS of *Bacillus* were significantly higher in EG than UG, SG, and MG (*p* < 0.05) at period B. *Bacillus* in MG exerted an important bioturbation effect on Daqu at periods C–E compared to UG (Figure 4C). For *Aspergillus*, we observed significant differences in the PS only during period E, where the PS of *Aspergillus* in the SG and MG groups were significantly higher than in the UG group, indicating that the perturbing effect of *Aspergillus* on the microbial community of HTD was mainly carried out in the E period (Figure 4D). *Pichia* were not significantly different throughout the fermentation stage in the three groups of fortified Daqu compared to UG (Figure 4E).

### 3.4 Analysis of differential species between groups in the fermentation stage of HTD

**Figure 5:**
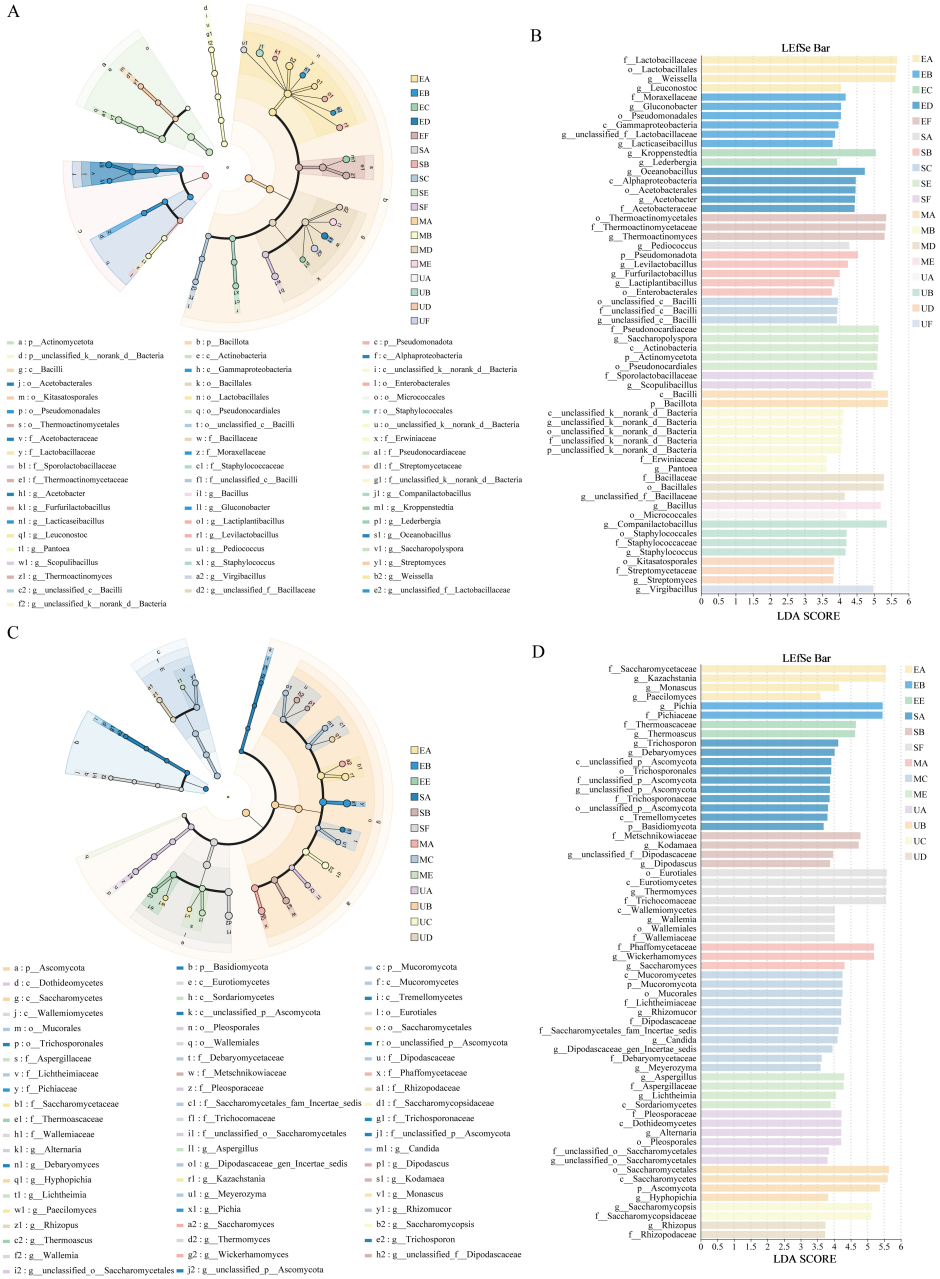
LEfSe multilevel discriminant analysis identified biomarkers in four HTD. Cladograms exhibited the evolutionary relationships of taxa, including bacterial genera **(A)** and fungal genera **(C)**. LDA scores (LDA > 3.5, *p* < 0.05) were computed for differentially abundant taxa between samples, with bacterial genera **(B)** and fungal genera **(D)** showing significant differences.

To investigate the impact of SFM biofortification on the variability and development of microbial taxa in HTD across different taxonomic levels, we applied LEfSe to identify biomarkers in the different groups. The LEfSe cladogram of bacteria indicated that the species differentiation of bacteria in EG and SG starts at the Class level and is higher at the Order level (Figure 5A). A total of 25 bacterial genera showed significant differences across the four HTD groups. Among them, 4, 10, 7, and 4 biomarkers at the genus level were identified in UG, EG, SG, and MG, respectively (*p* < 0.05, LDA score >3.5) (Figure 5B). Specifically, the UG group includes *Virgibacillus*, *Streptomyces*, *Staphylococcus*, and *Companilactobacillus*. The EG group includes *Thermoactinomyces*, *Acetobacter*, *Oceanobacillus*, *Lederbergia*, *Kroppenstedtia*, *Lacticaseibacillus*, *g__unclassified_f__Lactobacillaceae*, *Gluconobacter*, *Leuconostoc*, and *Weissella*. The SG group includes *Scopulibacillus*, *Saccharopolyspora*, *g__unclassified_c__Bacilli*, *Lactiplantibacillus*, *Furfurilactobacillus*, *Levilactobacillus*, and *Pediococcus*. The MG group includes *Bacillus*, *g__unclassified_f__Bacillaceae*, *Pantoea*, and *g__unclassified_k__norank_d__Bacteria*. The LEfSe cladogram for fungi indicated that the species differentiation of fungal communities from the Order level (Figure 5C). A total of 26 fungi genera showed significant differences across the four HTD groups. Among them, 6, 5, 8, and 8 biomarkers at the genus level were identified in UG, EG, SG, and MG, respectively (*p* < 0.05, LDA score >3.5) (Figure 5D). To elaborate, The UG group includes *Rhizopu*, *Saccharomycopsis*, *Hyphopichia*, *Saccharomycetes*, *g__unclassified_o__Saccharomycetales*, and *Alternaria*. The EG group includes *Thermoascus*, *Pichia*, *Paecilomyces*, *Monascus*, and *Kazachstania*. The SG group includes. Lastly, the MG group includes *Lichtheimia*, *Aspergillus*, *Meyerozyma*, *g__Dipodascaceae_gen_Incertae_sedis*, *Candida*, *Rhizomucor*, *Saccharomyces*, and *Wickerhamomyces.*

### 3.5 Co-occurrence patterns of microbial communities in four groups of HTD during the fermentation process

**Figure 6:**
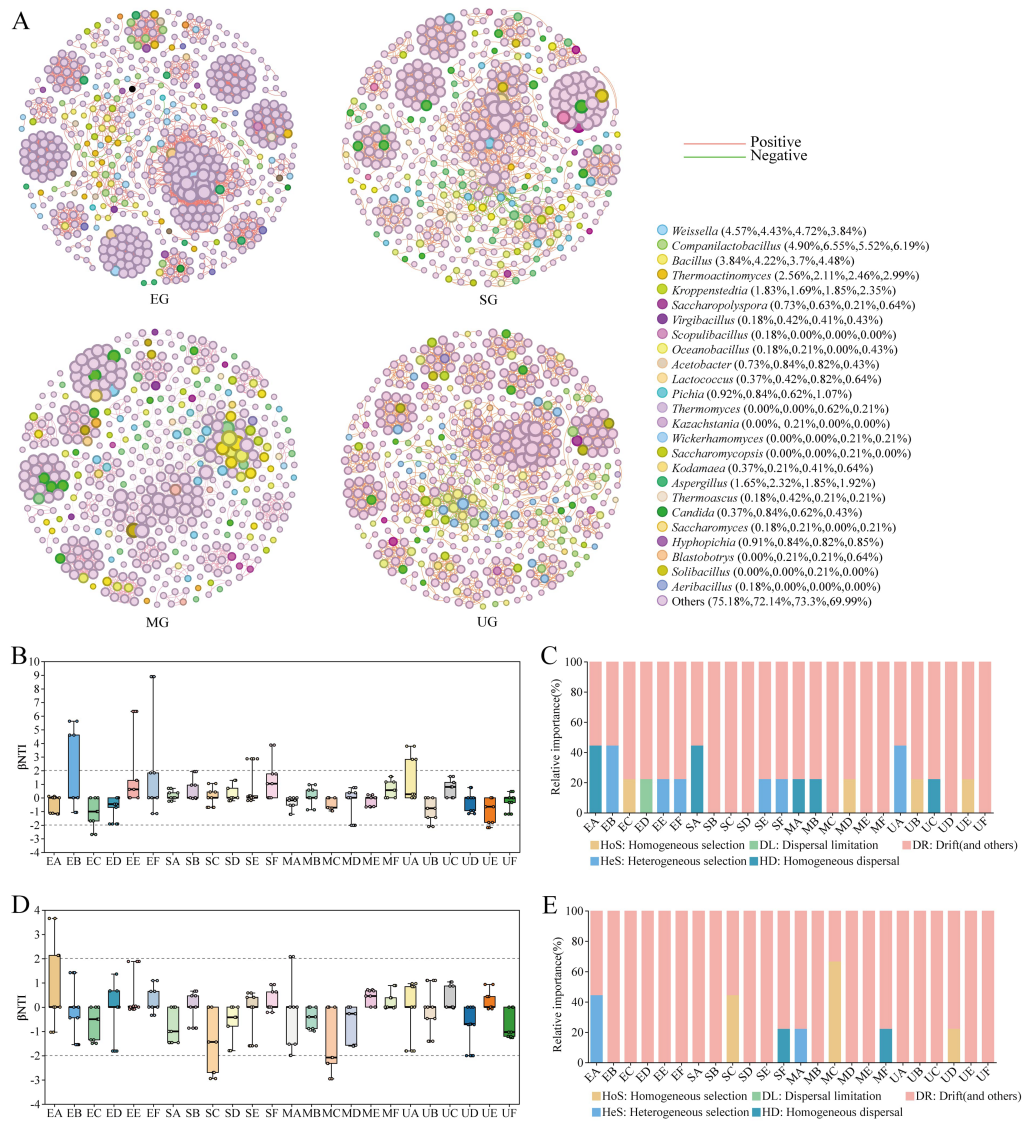
Co-occurrence patterns of microbial communities in four groups of HTD during the fermentation process **(A)**. Each node represents a single OUT, and the size of the node represents the relative abundance of the OUT. Each edge represents significant correlation (|r| > 0.8, *p* < 0.05), the line width represents the strength of correlation, and the red and green edges represent significant positive and negative correlation, respectively. Ecological analysis of the effect of different SFM on the community assembly during HTD fermentation, dynamics of βNTI for the bacterial **(B)** and fungal **(D)** communities. The relative importance of ecological processes to the assembly of bacterial **(C)** and fungal **(E)** communities.

The topological properties of the network were utilized to characterize the effects of the addition of different SFM on the interactions between the microorganisms of the HTD and on changes of the key taxa. We observed an increase in the number of nodes in the fortified HTD samples treated with various SFM. Specifically, the EG group comprised 548 nodes and 3,570 edges, the SG consisted of 474 nodes and 2,218 edges, and the MG encompassed 487 nodes with 2057 edges (Supplementary Table S4; Figure 6A). However, in the control group UG, only 469 nodes and 2,220 edges were observed. Interestingly, the addition of all three SFM resulted in an elevated proportion of positive links and a reduction in negative links within the HTD network. Specifically, the proportion of positive links rose to 99.86% for EG, 98.24% for SG, and 97.18% for MG, compared to the UG group, which exhibited a lower proportion of 96.94%. Meanwhile, we noted that the EG network exhibited higher values of avgK, Density, and avgCC compared to the UG network. In addition, within the EG network, *Weissella* demonstrated a strong positive correlation with *Blastobotrys*, and *Aspergillus* showed a strong positive association with both *Thermoactinomyces* and *Saccharomyces.* The proportion of positive links for EG, SG, and MG increased to 99.86, 98.24, and 97.18% compared to the UG group which had a proportion of 96.94%, respectively. This suggests that the addition of SFM promoted the positive correlation between microbiota in HTD. ‌Meanwhile, we observed that avgK, Density, and avgCC were higher in the EG network compared to the UG network. Furthermore, within the EG network, *Weissella* exhibited a strong positive correlation with *Blastobotrys*, whereas *Aspergillus* was strongly positively associated with both *Thermoactinomyces* and *Saccharomyces*. We also found that *Pichia* and *Aspergillus* were positively correlated with all other genera in SG network. ‌In the MG group, *Bacillus* exhibited a robust positive correlation with the genera *Solibacillus* and *Aeribacillus*, while demonstrating a notable negative correlation with *Weissella* and *Lactobacillus*. These findings support that inoculation of SFM biofortified HTD fermentation enhances the complexity and tightness of the microbial network within Daqu.

### 3.6 Different SFM and environmental factors influence the construction and assembly of microbial communities in the HTD fermentation stage

‌To elucidate the impact of various SFM on microbial community differentiation during the HTD fermentation stage, a null model was employed to evaluate the disparities in microbial community assembly patterns across the four Daqu groups. βNTI and RC_bray_ were utilized to quantify the effects of different SFM on the assembly patterns of HTD communities. The microbial community assembly of the four groups of HTD was mainly dominated by stochastic processes throughout the fermentation stage (|βNTI| < 2), indicating that the microbial communities were significantly below the phylogenetic turnover (Figures 6B,D). We found that different SFM exerted differential impacts on the microbial community assembly during the fermentation stage of Daqu (Figures 6C,E). Due to its relatively fixed position, DR dominated the assembly of bacterial and fungal communities in the four groups. Notably, in Biofortified Daqu groups (EG, SG, and MG), the initial assembly of bacterial communities was predominantly governed by HD limitation (RC_bray_ < − 0.95). In contrast, the untreated group (UG) demonstrated an initial bacterial community assembly primarily driven by HeS. Furthermore, throughout the fermentation process, the bacterial community structure in the UG group was predominantly influenced by HoS and HD. In comparison, among the fortified Daqu groups, EG exhibited a complex assembly pattern dominated by HeS, HoS, and DL. SG was dominated by HeS, and MG was dominated by HoS. For fungal community assembly, the initial assembly of EG and MG was also dominated by HeS. Throughout the fermentation stage, UG was also dominated by HoS only in period D. SG was also dominated by HoS and HD in periods C and F, respectively, and MG was also dominated by HoS only in period C.

**Figure 7:**
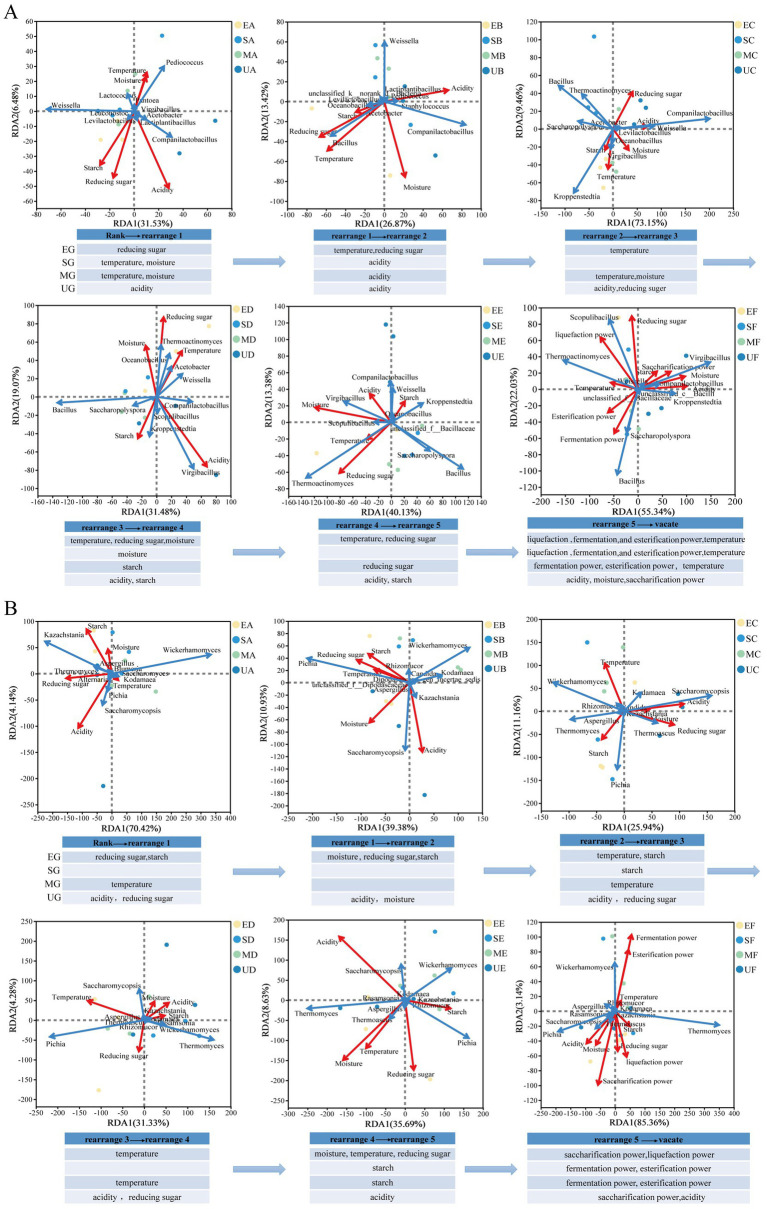
Redundancy Analysis between microbial community and physicochemical parameters based on the top 10 dominant microbes at genus levels during the Daqu fermentation phase. Different groups of samples are indicated by different coloured dots. The red arrows expressed physicochemical factors, and blue arrows stood for dominant microbes. The length of arrows determines the degree of importance. **(A)** bacteria. **(B)** fungi.

We employed redundancy analysis (RDA) by refining the study to different periods in the fermentation stage. The key factors driving the assembly of Daqu community were determined by combining the top 10 microorganisms of RA. The composition of the bacterial community in EG was primarily driven by temperature and reducing sugar content, whereas the fungal community was predominantly influenced by the interplay of temperature, starch, and reducing sugar content. The bacterial community in SG exhibited a strong dependence on moisture and acidity, with the fungal community demonstrating a notable correlation with starch content. The bacterial community in MG responded to a diverse array of environmental factors, while acidity emerged as the primary determinant of the fungal community (Figures 7A,B). In addition, at the end of the fermentation, *Virgibacillus* and *Kroppenstedtia* within the UG group exhibited positive correlations predominantly with acidity, moisture, and saccharification power. In contrast, the fungal community in this group demonstrated a more pronounced association with saccharification power. For the EG and SG, *Thermoactinomyces* showed a stronger correlation with liquefaction power, while the fungal community in the EG specifically displayed a heightened affinity for saccharification power. Notably, the fungal community in the SG group exhibited a significant positive correlation with both fermentation power and esterification power. *Bacillus* in the MG demonstrated a robust positive correlation with fermentation power, and the fungal community in this group also showed a notable association with liquefaction power, fermentation power, and esterification power.

### 3.7 Analysis of carbohydrate metabolic pathways of Daqu

**Figure 8:**
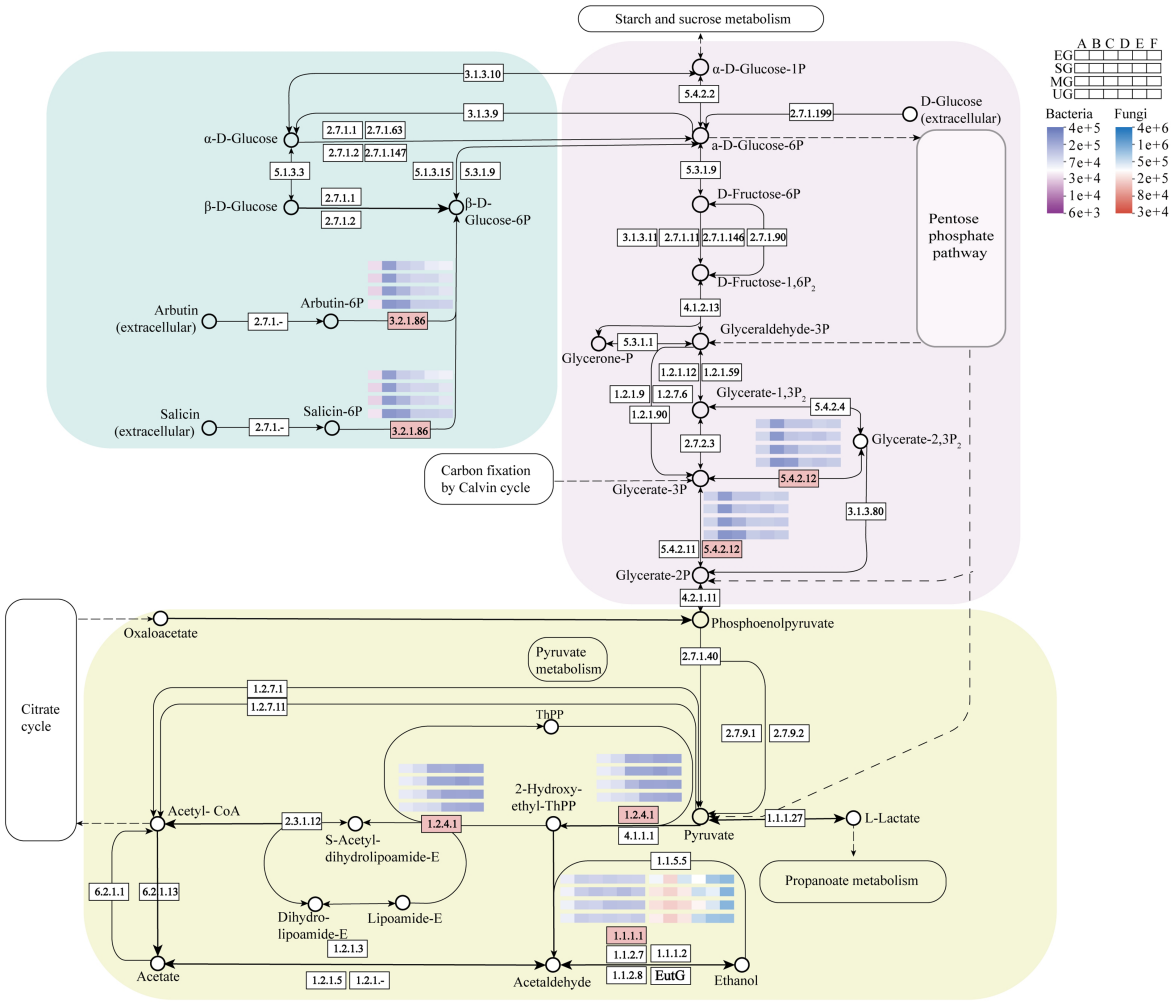
Starch and sucrose metabolism pathways in HTD, in which heatmaps show the abundance changes of four groups of HTD related functional enzymes. Pink boxes represent differentially expressed functional enzymes.

The gene expression of functional enzymes associated with starch and sucrose metabolic pathways during fermentation in the four HTD groups was predicted (Figure 8). 6-phospho-beta-glucosidase (EC 3.2.1.86) primarily converts non-sugar substances produced in the extracellular to *β*-D-Glucose-6P. In this work, we observed that (EC 3.2.1.86) expression was lower than control in EG, SG, and MG Daqu samples throughout the fermentation stage, with the most significant reduction in period C, which was 0.51, 0.50, and 0.29 folds lower than UG, respectively. In the glycolytic pathway, (EC 5.4.2.12) catalyzes the glycerate-2P and glycerate-3P reciprocal isomerization reactions in microorganisms. In the three groups of fortified Daqu samples, the gene expression of (EC 5.4.2.12) mainly showed a down-regulation trend. Especially in C period, it was reduced by 0.48, 0.47, and 0.28 folds compared with UG in EG, SG, and MG groups, respectively. Pyruvate dehydrogenase (EC 1.2.4.1) and ethanol dehydrogenase (EC 1.1.1.1) are important in the pyruvate metabolic pathway. Among them, gene expression of (EC 1.2.4.1) was significantly higher than that of the control group in all three groups of biofortified Daqu at period C after inoculation with SFM. Specifically, pyruvate dehydrogenase (EC 1.2.4.1) predominantly catalyzed the pyruvate oxidative decarboxylation reaction. In B-E periods, (EC 1.2.4.1) expression was higher in both EG and SG groups than in control UG, and notably in C period it was 0.92 and 1.15 folds higher in EG and SG than in UG, respectively. In contrast, the expression level in MG was significantly elevated during C-F periods compared to UG, with a 1.61-fold increase observed at the C period relative to UG. Ethanol dehydrogenase (EC 1.1.1.1) and L-lactate dehydrogenase (EC 1.1.1.27) further anaerobically metabolize 2-Hydroxy-ethyl-ThPP to ethanol and lactate. Ethanol dehydrogenase (EC 1.1.1.1) is a key enzyme in ethanol metabolism, with gene expression levels being significantly elevated in fungi compared to bacteria within the Daqu ecosystem. In bacteria, expression in EG did not differ much from UG, but was increased to 0.43-fold in E period in SG and just 0.26-fold in MG Daqu. However, in fungi, there was a large difference in the expression of (EC 1.1.1.1) in the four groups of Daqu from the C period onwards. The highest expression was found in EG, which was raised to 1.08-fold of UG in C period. In SG, there was little difference from UG except for the E period where it was reduced to 0.52-fold of UG. (EC 1.1.1.1) had the lowest expression in MG and was lower than UG in both C-E periods, with reductions of 0.15, 0.50, and 0.45 folds, respectively.

## 4 Discussion

‌The quality of sauce-aroma Baijiu is directly influenced by the HTD (Zhang et al., 2022). Therefore, to improve and enhance the quality of HTD, we have constructed three SFM and successfully developed three distinct varieties of biofortified Daqu in this work. Our findings revealed that all three biofortified Daqu significantly improved the liquefaction power. Furthermore, the introduction of SFM induced significant alterations in both the structure and function of the microbial community during the fermentation process. These changes are likely the primary contributors to the improved functionality and elevated quality of the fortified Daqu.

The liquefaction power of the Daqu reflects the ability of enzymes such as alpha-amylase in Daqu to liquefy starch (Xia et al., 2022b). The inoculation of SFM significantly enhanced the liquefaction power of the fortified Daqu, and the starch content during the C-F periods was consistently lower than that of UG, demonstrated that all three constructed SFM could enhance the hydrolysis and metabolic utilization of starch by Daqu microorganisms. Since the EG group was inoculated only with *E. amstelodami* synthetic flora, it was demonstrated that *E. amstelodami* synthetic flora significantly increased the liquefaction power of the Daqu. ‌As a probiotic, *E. amstelodami* exhibits robust enzyme-producing capabilities and possesses the ability to effectively biotransform a diverse array of substances, while also generating a spectrum of health-beneficial secondary metabolites (Gu et al., 2019; Li S. et al., 2024; Wang et al., 2024). The liquefaction power of both SG and MG was lower than that of EG, potentially due to the decreased inoculation percentage with the *E. amstelodami* flora. The diminished liquefaction power observed in MG may be ascribed to the inclusion of *Bacillus* species within the SFM, which contrasts with the results reported by Liu et al. (2024), who documented a notable enhancement in liquefaction power following the addition of *Bacillus* to Daqu. ‌ *Bacillus* species synthesize lipopeptides that exhibit antifungal properties, capable of interfering with and suppressing the growth of fungi, thereby restricting the proliferation of the fungal community within Daqu (Lax et al., 2019; Li Z. et al., 2023). Simultaneously, this may also be related to the fermentation of the Daqu in an open environment. The substantial enhancement in the fermentation power of SG is similar to the findings of Wang et al. (2023b), who employed *S. cerevisiae* in the preparation of fortified Daqu and demonstrated a notable increase in its fermentation power. ‌The MG group was also supplemented with yeast flora, however, the absence of a substantial enhancement in the fermentation power primarily stemmed from the relatively lower yeast inoculum compared to the SG group. The substantial elevation in esterification power observed in both the MG and SG groups suggested that the enzymes present within these two types of Daqu exhibit a marked catalytic effect on the production of esters, including ethyl hexanoate, ethyl acetate, and ethyl lactate (Fan et al., 2018). Controlling the saccharification power of ensures the stability of the quality of Sauce-aroma Baijiu, because the low saccharification force can ensure the production of Sauce-aroma Baijiu in multiple rounds (Tang P. et al., 2024). The substantial decrease in saccharification power in the SG group may have resulted from the inhibition of the growth of some glycosylase-producing microorganisms by the bioturbation effect of the SFM. After inoculation with SFM, we found that the changes produced in the physicochemical aspects of the Daqu were intimately associated with the enhancement of the functional strains of the microbial community, ultimately leading to enhanced enzyme functional properties of the Daqu. This shows that SFM biofortified Daqu utilize significantly more raw materials than traditional Daqu that rely on natural inoculation (Zhang et al., 2023), which is one of the effective ways to reduce costs. By selecting strains with high enzyme production and flavor synthesis to form functional modules can optimize Daqu quality, enhance fermentation stability, and shorten the fermentation cycle.

‌Significant variations in the composition of microbial communities can serve as an indicator for assessing the bioturbation effects of fortified Daqu, whose structural composition critically determines its brewing functionality (He et al., 2020; Zhu et al., 2023). Changes in the alpha-diversity index of the HTD after bioturbation by SFM indicated a change in the microbial community, which is similar to the results of previous studies (Li et al., 2017). This observation may be attributed to the intricate interactions between the SFM and the indigenous microorganisms within Daqu, which facilitate the periodic rearrangement of diverse microbial taxa, and this rearrangement is intricately linked to the enhancement of Daqu quality. ‌Following bioturbation by the SFM, the diversity and abundance of the bacterial community in the fortified Daqu were observed to be higher than those of the fungal community, a finding that is consistent with previous research results (Mu et al., 2023a). In the SG and MG groups, the SFM accelerated the succession rate of bacterial community, which proved that the reasonable design and addition of functional microflora to enhance the fermentation of Daqu can lead to the earlier formation of microbial and enzyme profiles. ‌Moreover, this contributes to the shortening of the production cycle for Daqu during winter, as Daqu produced in summer and autumn generally exhibits superior quality and a slightly shorter production cycle compared to that in winter (Fu G. M. et al., 2021; Liang et al., 2022). Based on the results of clustering analysis, we found that the inoculation with SFM significantly influences the differentiation and succession of bacterial and fungal communities during the fermentation stage, yet it does not alter the direction of fungal community succession. At the genus level, we have unveiled that the periods during which functional microorganisms in SFM exert their primary effects on HTD are not uniform. This suggests that it is feasible to regulate the quality of HTD by selectively adding functional microorganisms to the SFM according to production requirements.

We observed altered microbial community structure and abundance for all three fortified Daqu, which is consistent with previous reports (He et al., 2019b; Li et al., 2020). The RA of *Lactobacillus* was reduced in all three groups of fortified Daqu, which is similar to the findings of Pan et al. (2023). In our results, the EG group showed the best reduction of *Lactobacillus*. Given that elevated lactic acid levels can disrupt the proton balance across microbial cell membranes, leading to membrane damage and subsequent cellular impairment (Wei et al., 2023), so that the growth of other fermentation function microorganisms and sauce-aroma Baijiu production will be affected. Consequently, inoculation of *E. amstelodami* synthetic flora is an effective strategy to regulate lactic acid content during the production of sauce-aroma Baijiu. The higher RA of *Lactobacillus* in the SG and MG groups than in the EG group may be related to the addition of *S. cerevisiae* to the SFM, and previous studies have demonstrated that the interaction of microorganisms of *Lactobacillus* with *S. cerevisiae* improves the environmental adaptation and the biomass of *Lactobacillus* (Fan et al., 2020). The RA of *Weissella* was reduced in EG, SG, and MG in period C. Since previous studies have shown that *Weissella* and *Lactobacillus* are closely related (Teixeira et al., 2021), we hypothesized that the RA of *Lactobacillus* was first reduced in period B and then indirectly caused a reduction in the RA of *Weissella*. ‌SFM may also rapidly occupy ecological niches in the initial stages, thereby expanding its population dominance and constraining the establishment of later-arriving species (Stroud et al., 2024). Ecological niche occupation typically arises among species with substantial ecological niche overlap, and these microorganisms often possess similar functional genes, leading to analogous functionalities (Debray et al., 2021). Consequently, in this study, the inoculation of synthetic functional microbial consortia led to a marked enhancement in community function, despite perturbations in the community structure. ‌An elevated RA of *Bacillus* within the three fortified Daqu was conducive to enhanced production of hydrolytic enzymes and pyrazines (Liu et al., 2018; Tang et al., 2023). We observed a drastic decrease in fungal species during fermentation period B. In particular, the RA of *Thermomyces* decreased to less than 1%. This phenomenon was primarily attributed to substantial alterations in the fermentation microecological environment and was further influenced by the proliferation of bacterial species. The high proliferation rates of bacterial genera likely competed with fungi for shared ecological niches (Prosser et al., 2007).

‌The interactions among dominant species within the Daqu community, as well as the notable enrichment of differentiated microorganisms, are closely linked to the quality and stability of the brewing microecosystem (Ling et al., 2020). The inoculation with different SFM results in significant differences in the biomarkers of the enriched Daqu, which is likely a key determinant of the functional divergence observed in Daqu. We observed that the biomarker taxon *Thermomyces*, which was significantly enriched in the SG group, exhibits high thermal stability (Zhou et al., 2022), and *Lactococcus* was closely associated with the succession of microbial communities and the formation of flavor compounds during the Daqu fermentation process (Yang et al., 2023). We found that significant enrichment of *Bacillus* and other *Bacillus* species, *Saccharomyces* and *Wickerhamomyces* in the MG was closely related to yield, flavor and overall quality of sauce-aroma Baijiu (Niu et al., 2022). Brewing functional microorganisms such as *Weissella*, *Kroppenstedtia*, *Kazachstania*, *Issatchenkia*, *Pichia*, and *Thermoascus*, which are significantly enriched in EG, can promote the metabolism of Daqu substrates, so that the intermediate metabolites can be further converted into flavorful Baijiu brewing substances and their precursors (Yang et al., 2024). In addition, we found that many unknown and understudied microorganisms existed in the fermentation stage of the four groups of HTD, which increased the complexity of HTD fermentation and deserved further research.

Co-occurrence networks facilitate a deeper understanding of potential interactions among diverse microorganisms and unveil collaborative relationships within microbial communities (Barberán et al., 2012; Lei et al., 2025). Our results demonstrate that the introduction of SFM altered the ecological connectivity and complexity of microbial networks during the HTD fermentation process, increased the positive correlation between microorganisms. Therefore, microorganisms within the fortified Daqu may coexist through commensalism, cooperation, mutualism, and syntrophy, thereby promoting microbial community stability (Li M. et al., 2024). The MG group demonstrated a higher proportion of negative interactions compared to the EG and SG, suggesting a more intense competition among microorganisms in the biofortified Daqu of the MG group for environmental ecological niches and the limited resources generated through microbial metabolism. The highest network stability observed in the EG group suggests a significant association between *E. amstelodami* and the stability of the Daqu microbial community, necessitating further investigation into the underlying mechanisms of this relationship. The enhanced microbial network stability implies an increased resilience of the Daqu microbial community to environmental disturbances during the fermentation process (Zhao et al., 2023).

The bacterial community assembly of EG was most affected by SFM, suggested that the *E. amstelodami* flora could significantly influence the dynamics and function of microbial communities. The improved enzyme function of SG indicated that the inoculation of *E. amstelodami* flora and yeast flora at a ratio of 1:1 was the optimal combination to improve the quality of HTD. It also lays the theoretical and practical foundation for synthesizing functional microflora to enhance the quality of Daqu. The primary factor accounting for the community disparities observed in EG, SG, and MG was the inoculation with distinct SFM, as the initial assembly of the Daqu community was significantly affected by SFM. The combination of various SFM with abiotic factors induces distinct bioturbation effects on microbial community succession, modulating the interactions and metabolic activities within the community, and ultimately shaping diverse microecological environments. Prior research has demonstrated that the diversity and intricacy of interspecies interactions among microflora affect the dynamics and function of microbial communities (Blanchard and Lu, 2015; Ruiz et al., 2023). No significant temperature variation was observed among the four Daqu groups, and all of them were able to reach more than 60°C before the first turnover. Combined with the high humidity environment at this time, the Maillard reaction occurred, leading to the formation of aromatic hydrocarbons or their precursors (Hu et al., 2021). In our work it was found that temperature and moisture had the most significant effect on the driving and assembly of the EG microbial community and the SG bacterial community, respectively. This enabled Daqu to develop a unique microbial community structure and enzyme system under the environmental stress of high temperature and high humidity (Huang et al., 2017b). The acidity of EG, SG, and MG were all lower than that of UG, which was similar to the results of the previous study (Mu et al., 2023b), and this was mainly caused by the fact that functional microorganisms in SFM first occupied the ecological niche in the early fermentation stage and influenced the microorganisms to produce organic acids. In this work, we have only investigated the effect of SFM on the solid-state fermentation process of HTD. We expect to further quantify of the contents of ethanol, esters, and organic acids using techniques such as HPLC and HS-SPME-GC/MS will be crucial for elucidating the endogenous regulatory mechanisms of HTD flavor modulated by the addition of SFM. Concurrently, we plan to apply SFM in the actual production of Baijiu, with a focus on examining and evaluating its impacts on the quality and yield of the final product.

The gene expression profiles of the enzymes in the starch and sucrose metabolic pathways were significantly altered in the fortified Daqu, especially the gene expression of both (EC 1.2.4.1) and (EC 1.1.1.1) within the pyruvate metabolic pathway was up-regulated. Increased expression of these two enzymes enhanced the potential ability of the microbial community in the fortified Daqu to utilize energetic substances such as starch for metabolism and energy conversion, to promote ethanol metabolism, and to produce esters and alcohols (Borzova et al., 2023). This facilitates fermentation efficiency and promotes the production of flavor compounds.

## 5 Conclusion

In this work, we confirmed that the inoculation of SFM ‌enhanced‌ the enzymatic ‌activity‌ of biofortified Daqu and ‌improved‌ its liquefaction, fermentation, and esterification power ‌variably‌. Among them, ‌*E. amstelodami* ‌demonstrated a marked stimulatory effect‌. Furthermore, the improvement of the quality of biofortified Daqu by SFM leads to alterations in the structure and composition of the microbial community during the fermentation process. Additionally, the number of nodes and the proportion of positively correlated edges in the microbial network during the fermentation process of the fortified Daqu have significantly increased. In conclusion, the utilization of SFM to fortify Daqu fermentation is an effective approach to enhance the quality of Daqu. Some of the results of this work can offer certain insights for the rational utilization of functional microorganisms to construct SFM for improving the quality of HTD for sauce-aroma Baijiu.

## Data Availability

The datasets presented in this study can be found in online repositories. The names of the repository/repositories and accession number(s) can be found in the article/Supplementary material.
